# Modeling Viral Capsid Assembly: A Review of Computational Strategies and Applications

**DOI:** 10.3390/cells13242088

**Published:** 2024-12-17

**Authors:** Wenhan Guo, Esther Alarcon, Jason E. Sanchez, Chuan Xiao, Lin Li

**Affiliations:** 1 Department of Physics, University of Texas at El Paso, El Paso, TX 79968, USA; wguo2@utep.edu; 2 Department of Chemistry and Biochemistry, University of Texas at El Paso, El Paso, TX 79968, USA; ealarcon5@miners.utep.edu; 3 Department of Computational Science, University of Texas at El Paso, El Paso, TX 79968, USA; jesanchez6@miners.utep.edu

**Keywords:** viral capsid assembly, computational methods, molecular dynamics simulations, coarse-grained models

## Abstract

Viral capsid assembly is a complex and critical process, essential for understanding viral behavior, evolution, and the development of antiviral treatments, vaccines, and nanotechnology. Significant progress in studying viral capsid assembly has been achieved through various computational approaches, including molecular dynamics (MD) simulations, stochastic dynamics simulations, coarse-grained (CG) models, electrostatic analyses, lattice models, hybrid techniques, machine learning methods, and kinetic models. Each of these techniques offers unique advantages, and by integrating these diverse computational strategies, researchers can more accurately model the dynamic behaviors and structural features of viral capsids, deepening our understanding of the assembly process. This review provides a comprehensive overview of studies on viral capsid assembly, emphasizing their critical role in advancing our knowledge. It examines the contributions, strengths, and limitations of different computational methods, presents key computational works in the field, and analyzes milestone studies that have shaped current research.

## 1. Introduction

Historically, viruses were defined as small infectious agents capable of passing through 0.2 µm filters. Many viruses consist of nucleic acid surrounded by protein shells, called capsids, that encapsulate their genomic material (either DNA or RNA) [1,2,3,4,5]. Some viruses also possess lipid envelopes embedded with glycoproteins. As well as protecting the viral genomes, the capsid performs other critical functions such as facilitating host recognition and delivering the viral genome into host cells. Due to the coding limitations of their small genomes, many virus capsids are assembled from repeating building blocks following symmetrical patterns. For instance, numerous spherical viruses use icosahedral symmetry to form 20-faced polygonal particles [6,7,8,9]. Covering the icosahedral surface with repeating building units requires special geometric shapes [10]. More than one-third of viruses infecting all three domains of life utilize a distinctive protein motif called the jelly-roll fold (JRF) [11,12]. The JRF meets this requirement by forming a wedge shape from two beta-sheets, each consisting of four beta-strands [13]. In recent decades, many giant viruses with a physical size greater than 0.2 µm have been discovered, challenging the original definition of a virus [14,15,16]. Although certain giant viruses have genomes larger than those of small bacteria, their capsids still follow symmetrical assembly, comprising thousands of repeating units called capsomers. However, the process by which these large numbers of capsomers accurately assemble into the capsid remains poorly understood. The size and complexity of giant viruses have posed significant challenges in structural studies [13].

During viral infection, the assembly process is important for producing infectious progeny virus particles. The assembly process of viral capsids is complex and highly regulated. This process involves the coordinated expression of various components and is influenced by various factors, including protein–protein interactions, electrostatic forces, and various environmental conditions [9,17,18,19]. Back in 1962, D. L. D. Caspar and A. Klug studied the construction of regular viruses from a physical principles perspective, which later developed into the Caspar–Klug theory (CK Theory) [20]. Caspar has a long-standing and influential history in the study of viral structure and capsid assembly. His early work, particularly on the polyomavirus capsid protein VP1 and the tobacco mosaic virus, elucidated the principles of viral particle assembly and the self-assembly mechanisms of viral capsid proteins [21,22,23,24,25,26]. Zlotnick’s research group began studying virus capsid assembly in the 1990s, with a particular focus on the assembly of the hepatitis B virus capsid and has achieved significant advancements in this field [9,27,28,29,30,31,32]. He developed a classic kinetic model that is widely used to study the process of viral capsid assembly [9]. The model, based on simple reaction kinetic equations, can describe the process by which capsid proteins self-assemble into stable shells under specific conditions, revealing key intermediates and reaction pathways in the assembly process. Since the 2000s, Roya Zandi’s group, from the field of physics, has focused on studying the impact of electrostatic and entropic mechanisms on the stability of viral structures and the self-assembly process, investigating the origins of capsid symmetry, packaging mechanisms, and mechanical properties [33,34,35,36,37]. During the same period, Twarock and her team, from a biological perspective, use geometric models to study the assembly mechanisms of viral capsids and genomes, providing new insights into understanding virus structure and developing antiviral strategies [38,39,40].

The study of viral capsid assembly through computational approaches has been a significant area of interest in virology and structural biology. Numerous retrospective studies have emerged in the past two decades, focusing on the computational methods used for understanding viral capsid assembly. In 2008, Dr. Robert L. Garcea’s laboratory reviewed the biological constraints on viral assembly modeling, identifying key variables that could be incorporated to enhance the real-world relevance of mathematical models for viral assembly [41]. In 2014, Michael F. Hagan discussed the capabilities and limitations of various approaches, ranging from equilibrium continuum theories to molecular dynamics simulations, in understanding virus assembly. His work provided an overview of key conclusions from these models, covering topics such as the assembly of empty viral shells, assembly around single-stranded nucleic acids, and assembly around synthetic polymers or charged nanoparticles for nanotechnology or biomedical applications. Hagan highlighted instances where modeling has driven experimental breakthroughs and suggested ways to strengthen the connection between modeling and experimentation [42]. In the same year, Hagan also reviewed the physical principles governing capsid assembly both within host cells and in vitro, identifying areas of virus assembly that were likely to receive significant attention [19]. In 2016, Hagan and Zandi reviewed the coarse-grained modeling of virus assembly. Same year, Mark S.P. Sansom and Tyler Reddy discussed the use of molecular dynamics simulations in studying virus capsid assembly, highlighting the computational challenges in simulating complex viral membranes [43]. In 2017, Toru Ekimoto and Mitsunori Ikeguchi used multiscale MD simulations, combining efficient sampling techniques and coarse-grained (CG) approaches with all-atom MD simulations, to review two examples of rotary motor proteins analyzed through free energy landscape (FEL) analysis and CG-MD simulations [44]. In 2020, Chuan Xiao’s group reviewed recent assembly models and supporting experimental observations of Nucleocytoviricota Viruses (NCVs) [13]. In the same year, Eric R. May and Asis K. Jana reviewed studies on virus capsids using cryo-electron microscopy and molecular dynamics simulations, furthering the understanding of their physical–chemical properties and biological impact [45]. In 2021, Jodi A. Hadden-Perilla’s group reviewed the progress and future prospects of molecular dynamics simulations [46]. Recent developments in viral architecture, particularly in computational studies, have begun to challenge the traditional Caspar–Klug model, which has long served as the foundation for understanding icosahedral virus structure. Advances in high-resolution cryo-electron microscopy and molecular dynamics simulations have revealed structural variations and asymmetries in viruses like HIV-1 and human papillomavirus (HPV) that deviate from the strict symmetry predicted by the Caspar–Klug model. Computational models have further demonstrated that viral capsids can exhibit non-classical assembly pathways and dynamic structural adaptations in response to environmental factors, such as RNA packaging and molecular crowding. These findings suggest that viral assembly is a more flexible and heterogeneous process than previously thought, with significant implications for the design of virus-like particles and custom nanocages in nanotechnology. This evolving understanding of viral architecture not only enhances our knowledge of virus biology but also opens new avenues for developing innovative antiviral strategies and nanomaterials.

Building on the previous discussion, computational approaches, such as molecular dynamics (MD) simulations, stochastic dynamics simulations, coarse-grained (CG) models, and electrostatic analysis, have become powerful tools for studying and elucidating the principles that govern capsid assembly. These methods are widely utilized across various biological subfields due to their distinct strengths and limitations, making them well-suited for different aspects of viral capsid assembly and other complex biological processes [47,48,49]. Molecular dynamics (MD) simulations provide detailed, atomistic insights into molecular interactions and dynamic processes, making them highly accurate but computationally intensive, which limits their application to smaller systems or shorter timescales. Stochastic dynamics simulations use random sampling techniques to explore the configurational space of a system, making them effective for exploring the configurational space of complex systems and studying their statistical mechanical properties, but they often sacrifice dynamic information due to their focus on equilibrium states. Coarse-grained (CG) models reduce computational complexity by simplifying atomistic details, enabling the study of larger systems and longer timescales, though at the cost of losing some molecular detail. Electrostatic analysis is crucial for understanding interactions like protein-nucleic acid binding, offering insights into key molecular forces, but it often requires simplifications that may overlook finer structural details. Additionally, other computational methods, such as lattice models, hybrid methods, and machine learning techniques, have expanded the toolkit for studying viral assembly. Lattice models provide a simplified yet powerful means to explore general assembly principles. Hybrid methods combine different approaches to leverage their respective strengths. And machine learning techniques analyze large datasets to uncover patterns and predict assembly outcomes.

**Figure 1:**
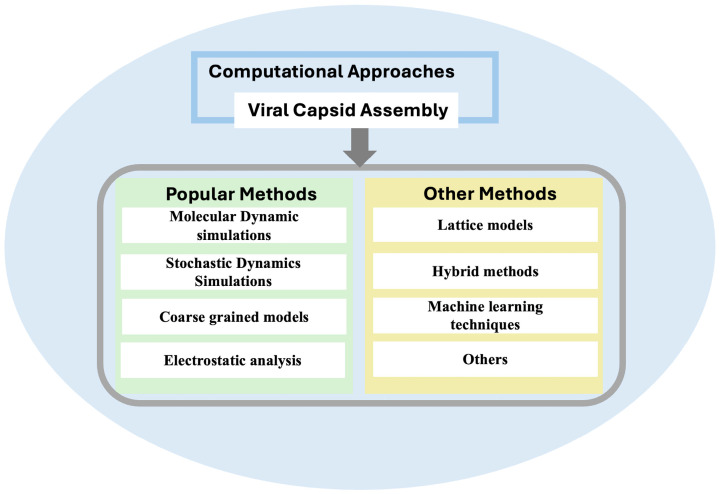
Computational approaches for understanding viral capsid assembly.

The integration of these computational methods also has significantly enhanced our understanding of viral capsid assembly, providing detailed insights into the molecular interactions and mechanisms that drive this complex process. By utilizing the strengths of each approach, researchers have been able to model the dynamic behaviors, statistical properties, and structural features of viral capsids with increasing accuracy. These advancements not only shed light on the fundamental principles of virus assembly but also pave the way for practical applications in biomedical and nanotechnology fields. Understanding the viral capsid assembly process through these methods is poised to drive advancements in various domains. These include the development of innovative antiviral therapies, repurposing viruses for drug delivery, and synthesizing self-assembling nanostructures. This paper reviews the advanced computational tools that have been utilized to elucidate the mechanisms of virus assembly, highlighting their contributions, advantages, and limitations in advancing our knowledge of viral capsid assembly. To accommodate space limitations, we limit our discussion to primary viral capsid assembly computational studies and categorize them into five areas: molecular dynamics (MD) simulations, stochastic dynamics simulations, coarse-grained (CG) models, electrostatic analysis, and other methods. The schematic diagram of the method is presented in Figure 1, with further details provided in the subsequent sections. We have made every effort to comprehensively cover the relevant literature in this review. However, given the breadth of research in this field, we may have inadvertently overlooked some important contributions. We apologize for any omissions and appreciate your understanding.

## 2. Molecular Dynamic Simulations

Molecular dynamics (MD) emerged in the late 1950s and early 1960s, with Berni Alder and Thomas Wainwright first using MD simulations in 1957 to study the dynamical behavior of simple hard-sphere liquids. In 1964, Aneesur Rahman further advanced MD techniques by applying them to the molecular dynamics of liquid argon. With revolutionary advances in computer technology and algorithmic improvements, MD has since the 1970s become widely used to study the structure and dynamics of macromolecules, such as proteins and nucleic acids [50,51,52,53,54]. MD simulations provide detailed atomic-level insights and are commonly employed to study biologically important macromolecules and their environments. They play a crucial role in investigating the interactions and dynamics involved in capsid assembly.

By numerically solving Newton’s equations of motion (Equation (1)) for particles in the system, MD simulations capture the conformational changes and assembly pathways of capsid proteins over time, enabling the derivation of key kinetic and thermodynamic properties. According to Newton’s second law, the force $F_{i}$ acting on a particle i of mass $m_{i}$ is related to its acceleration $a_{i}$ as follows:

(1) $$F_{i}=m_{i}a_{i}=m_{i}\frac{d^{2}r_{i}(t)}{dt^{2}}.$$

where $r_{i}\left(t\right)=(x_{i}\left(t\right),\;y_{i}\left(t\right),z_{i}(t))$ is the position vector of *i*th particle. To numerically solve the equations of motion, MD simulations employ integration schemes that approximate the continuous motion of particles over discrete time steps. One commonly used method is the Verlet algorithm, which updates the positions $r_{i}$ of the particles based on their positions at previous time steps and the forces acting upon them:

(2) $$r_{i}\left(t+∆t\right)≅2r_{i}\left(t\right)-r_{i}\left(t-∆t\right)+\frac{F_{i}\left(t\right)}{m_{i}}∆t^{2}.$$

The equation forms the computational foundation of molecular dynamics simulations, enabling the exploration of molecular systems at atomic resolution over time.

Newton’s second law is the core equation in molecular dynamics simulations; it describes how a particle’s motion evolves over time, while a force field provides the potential energy function ${U(r}_{i})$ that describes the interactions between atoms or molecules in a system. The force acting on each particle can be determined by taking the gradient of this potential energy function.

(3) $$F_{i}={-\nabla r}_{i}{U(r}_{i})=-\left(\frac{\partial U}{\partial x_{i}},\frac{\partial U}{\partial y_{i}},\frac{\partial U}{{\partial z}_{i}}\right),$$

where ${U(r}_{i})$ represents the total potential energy of the system, typically a sum of various interaction potentials, including bond stretching, bond angles, dihedral angles, van der Waals forces, and electrostatic interactions. $\nabla r_{i}$ denotes the gradient with respect to the position $r_{i}$ of particle i. A typical force field, used in the simulations of biosystems, takes the following form:

(4) $${U(r}_{i})=\sum_{bonds}\frac{a_{i}}{2}(l_{i-}l_{i0}{)}^{2}+\sum_{angles}\frac{b_{i}}{2}(\theta_{i-}\theta_{i0}{)}^{2}+\;\sum_{torsions}\frac{c_{i}}{2}[{1+cos(n\omega }_{i-}\gamma_{i})]+\;\sum_{atom\;pairs}4\epsilon_{ij}[(\frac{\sigma_{ij}}{r_{ij}}{)}^{12}-(\frac{\sigma_{ij}}{r_{ij}}{)}^{6}]+\sum_{atom\;pairs}k\frac{q_{i}q_{j}}{4\pi \epsilon_{0}r_{ij}}.$$

The first three terms represent the bonded interaction potentials: bond stretching potential, angle bending potential, and dihedral/torsion potential. The last two terms represent the nonbonded interaction potentials: van der Waals potential and Coulombic/electrostatic potential. In the first two terms, $l_{i}$ represents the bond lengths, $\theta_{i}$ represents the bond angles, and their respective equilibrium values are $l_{i0}$ and $\theta_{i0}$. The force constants are denoted by $a_{i}$ and $b_{i}$. In the third part, rotations around the chemical bond are characterized by periodic energy terms, with the periodicity determined by n and the heights of the rotational barriers defined by $c_{i}$. ω is the dihedral angle, γ is the phase angle. In the fourth term of Wan der Waals repulsive and attractive (dispersion) interatomic forces, $\epsilon_{ij}$ represents the well depth, indicating the strength of the attraction between atoms, while $\sigma_{ij}$ is the distance between two atoms when the potential energy is zero. $r_{ij}$ is the actual distance between atoms i and j. The last term is the Coulomb electrostatic potential. which can be influenced by specific environmental effects. These effects can be accounted for by properly adjusting the partial charges $q_{i}$ and the effective value of the constant k, as well as the van der Waals parameters $\epsilon_{ij}$ and $\sigma_{ij}$. $r_{ij}$ is the distance between two particles (i < j), $\epsilon_{0}$ is the permittivity of free space (dielectric constant).

Molecular dynamics (MD) simulations have become an indispensable tool for studying the intricate process of viral capsid assembly, offering a detailed and dynamic view that complements experimental methods. The complementary insights include the forces ruling viral assembly, stability, and dynamics. Notable milestone works include the simulation of the complete assembly of small icosahedral viruses and the study of protein–protein interactions in the capsid, which have provided critical insights into the mechanisms of capsid formation and stability [45,55,56,57,58]. One of the key strengths of MD in this context is its ability to provide high-resolution temporal and spatial data, allowing researchers to observe the self-assembly process in real time. Through these simulations, it is possible to identify intermediate structures, understand the sequence of events leading to capsid formation, and pinpoint the role of specific amino acids in the stability and integrity of the capsid. These insights are often difficult, if not impossible, to capture through experimental techniques alone, making MD simulations particularly valuable for exploring the molecular mechanisms underlying viral assembly. However, MD simulations are not without their limitations. Molecular dynamics (MD) simulations are highly resource-intensive, demanding substantial computational power and time. This limitation restricts their application to modeling very large systems or simulating processes that unfold over extended timescales, such as the full assembly of a viral capsid or its interactions with the host environment. While most simulations currently operate within a nanosecond to microsecond timescales, many crucial biological processes, like protein folding or large-scale conformational changes, occur over much longer durations, posing a considerable challenge for MD simulations. Despite these limitations, MD simulations play a crucial role in complementing experimental studies. They offer a level of molecular detail that is difficult to achieve with techniques such as X-ray crystallography or nuclear magnetic resonance (NMR), which often provide static snapshots rather than dynamic processes. By integrating MD simulations with experimental data, researchers can gain a more comprehensive understanding of viral capsid assembly, validating theoretical models and exploring scenarios that may not be feasible to recreate experimentally. This synergy between MD simulations and experimental approaches continues to drive forward our understanding of viral behavior, making it a vital component in the study of virology and the development of antiviral strategies.

The field has seen substantial progress since 1998, when a computational model was first established to describe the kinetics of icosahedral viral capsid self-assembly [55]. In 2006, MacPherson, Schulten, and collaborators developed an all-atom model for the satellite tobacco mosaic virus (STMV). Using MD simulations, they investigated the structural stability of the entire virus, the empty capsid, and the unencapsidated RNA genome. They analyzed key physical properties such as electrostatic potential, radial distribution of viral components, and correlated motion patterns, discussing their implications for the virus’s assembly and infection mechanisms. Their work provides critical insights into viral stability and dynamics [59]. In 2013, Klaus Schulten, Christopher Aiken, and Peijun Zhang’s lab established the mature HIV-1 capsid structure by cryo-electron microscopy and all-atom molecular dynamics. The complete atomic HIV-1 capsid model provides a platform for further studies of capsid function and targeted pharmacological intervention [60]. In 2015, K. Schulten and colleagues established the first all-atom model of an immature retroviral lattice of the Rous sarcoma virus (RSV) using MD simulations and in vitro assembly techniques, which was a milestone in understanding immature viral assembly stages [56]. The following year, Deborah G. Evans and collaborators employed multi-scale computational methods, including MD, random sampling, and electronic structure calculations, to study the interactions between oligomeric conjugated polyelectrolytes (OPEs) and the MS2 capsid. Their findings provided strategies for enhancing viral capsid disruption, crucial for targeting the protein coats of icosahedral-based viruses [61]. In 2017, Sergio Pantano’s group introduced an explicit water model for double/triple-scale MD simulations, making it possible to combine popular atomistic parameters with the coarse-grained (CG) water used by the SIRAH force field, thus enhancing the feasibility of simulations on desktop computers [62]. That same year, Juan R. Perilla and Klaus Schulten investigated the chemical–physical properties of the human immunodeficiency virus type 1 (HIV-1) capsid from over 1 μs all-atom molecular dynamics simulations, including its electrostatics, vibrational and acoustic properties, and the effects of solvent (ions and water) on the capsid [63]. In 2018, Hadden’s group used all-atom molecular dynamics simulations of the hepatitis B virus (HBV) capsid to reveal insights into its biological function and the limits of cryo-EM resolution. By investigating the capsid without symmetry bias, they were able to study its flexibility, confirming its propensity for asymmetric distortion. This work provides functional insights beyond the reach of experimental methods and highlights the importance of considering asymmetry in structural studies of icosahedral virus capsids [64]. Lin Li’s lab, in 2019, utilized MD simulations to identify four binding modes between dengue heterotetramers, which repeat periodically throughout the virus capsid, providing insights into the assembly mechanism of the dengue virus [65]. Lizabeth E. Jefferys and Mark S. P. Sansom’s 2019 book described three main areas of computational virology: small simulation systems, computational studies of viral capsid assembly and genome encapsidation, and developments in simulating entire viral particles, highlighting the breadth of computational approaches in the field [66]. The same year, MD simulations were used to simulate potential novel complexes of the trimodular ankyrin repeat protein (Ank1D4), aimed at inhibiting the N-terminal domain capsid protein (NTDCA) of HIV-1 during virus assembly and budding [67]. Yinghao Wu and his colleagues generated a multiscale computational framework by using rigid body-based diffusion, the residue-based kMC algorithm, and all-atom DMD simulations to simulate the self-assembly of the virus-like particle bacteriophage MS2 in 2019. They suggested that the alternate growth of viral capsids through heterotypic dimer interactions dominates the assembly pathways [68]. In 2020, Eric R. May and Asis K. Jana reviewed studies on icosahedrally symmetric virus capsids, emphasizing the use of cryo-electron microscopy (cryo-EM) and MD simulations to uncover asymmetric aspects crucial for understanding viral physical–chemical properties [45]. In 2021, Bo Chen’s lab combined MD simulations (NAMD) with solid-state NMR and prior cryoelectron tomography restraints to study retroviral capsid proteins (CAs) in polymorphic capsids, extending our understanding of capsid assembly modulation [57]. Szilard N. Fejer and Janos Szoverfi in 2022 evaluated the structural stabilities of different salt-stable cowpea chlorotic mottle virus (ss-CCMV) dimers, using MD simulations with implicit solvent models to determine conditions leading to their dissociation [69]. Dr. Carlos E. Catalano’s lab used dynamic methods to investigate the structure and biophysical properties of a soluble, assembly-deficient phage lambda major capsid protein, MCP (W308A) [70].

In the last five years, MD studies in virus capsid assembly have focused extensively on Hepatitis B Virus (HBV) capsids, a key drug target for viral hepatitis. All-atom molecular dynamics simulations of an intact AT130-bound HBV capsid reveal that the compound increases spike flexibility and improves recovery of helical secondary structure in the spike tips, aiding in combating viral hepatitis [58]. Computational models have also provided insights into the binding characteristics between HBV proteins and pyrrole-scaffold inhibitors, guiding the identification of novel potent compounds [71]. James C. Gumbart’s group reported novel capsid assembly modulators (CAMs) discovered based on biophysics approaches, which combine docking, MD simulations, and a series of assays with a particular emphasis on biophysical experiments [72]. Building on such integrative efforts, enhancing the synergy between computational predictions and experimental validation will be essential for generating even more robust and impactful insights in the field.

## 3. Stochastic Dynamics Simulations

Stochastic simulation methods are a powerful class of computational tools used to study and analyze the behavior and properties of complex systems. Unlike traditional deterministic methods, stochastic simulation methods introduce randomness to explore the possible states or trajectories of a system, making them particularly effective in dealing with high-dimensional spaces, complex interactions, and nonlinear dynamics. In many scientific fields—especially in statistical physics, chemistry, biology, and materials science—the behavior of complex systems is often difficult to fully understand and predict through analytical methods or experimental techniques alone. In such cases, stochastic simulation methods offer an effective alternative, allowing researchers to explore equilibrium properties, dynamic processes, and behavior patterns under multi-body interactions through numerical simulations. The importance of stochastic simulation methods lies not only in their ability to tackle analytically intractable problems but also in their flexibility and versatility. Monte Carlo (MC) and Brownian dynamics (BD) are two primary forms of stochastic simulations. While they differ in their specific algorithms and application scenarios, they both share the common principle of utilizing stochastic processes. The MC method utilizes random sampling to calculate thermodynamic averages of a system, making it widely applicable in equilibrium studies. Meanwhile, Brownian dynamics (BD) simulates the random motion of particles to study their dynamic behavior in fluids, particularly suited for investigating long timescale interactions between molecules.

MC simulations are a powerful computational technique widely used to explore the thermodynamic and structural properties of complex systems. Unlike molecular dynamics (MD), which explicitly models the time evolution of a system, MC simulations rely on random sampling methods to explore the configurational space of particles or molecules. The core of the MC algorithm involves generating a series of random moves within the system, where each move represents a potential change in configuration, such as the rotation around a bond in a protein structure. These moves are then evaluated based on their impact on the system’s potential energy. Although MC simulations are often more efficient than MD simulations, their efficiency can still be significantly impacted in very large systems. MC methods typically require a substantial number of iterations to achieve adequate sampling, especially in high-dimensional systems, which can result in a considerable increase in computational time. In the widely used Metropolis MC algorithm, a move that lowers the system’s potential energy is always accepted, while a move that increases the potential energy is accepted with a probability determined by the Boltzmann factor. This approach allows MC simulations to efficiently sample a wide range of configurations, making them particularly useful for studying systems where direct simulation of dynamics, as in MD, would be computationally prohibitive. MC simulations have been instrumental in providing insights into the thermodynamics and kinetics of the self-assembly process of viral capsid assembly. By utilizing random sampling techniques, MC simulations can efficiently explore the vast configurational space of capsid proteins, identifying the most stable configurations and the pathways leading to their formation. This capability is especially valuable given the stochastic nature of capsid assembly, where proteins must navigate a complex energy landscape to form a stable and functional structure. Early MC simulations of capsid assembly often employed lattice-based models, which, despite their simplicity, offered fundamental insights into the principles governing capsid formation. These models helped to identify key factors such as the role of geometric constraints and protein–protein interactions in driving the assembly process. More recent advances in MC simulations have moved beyond lattice-based approaches, incorporating more realistic representations of protein–protein interactions and geometries. These non-lattice MC simulations have provided a deeper understanding of the assembly pathways, identifying energy minima corresponding to stable capsid structures and offering statistical insights into the factors that influence the efficiency and accuracy of the assembly process. However, it is important to note that while MC simulations excel in sampling large systems and capturing the statistical nature of assembly processes, they rely on simplified models and potential energy functions. This reliance can sometimes limit their accuracy in capturing detailed atomic interactions and dynamic behaviors, which are better addressed by methods like MD. Nonetheless, MC simulations remain a crucial tool in the study of viral capsid assembly, complementing other computational and experimental approaches to unravel the complexities of this vital biological process. In addition to conformational sampling, MC can also be used as a global optimization method, where the goal is to find a global minimum of the potential energy—a critical and challenging problem in protein structure determination. The MC optimization protocol is particularly effective when combined with simulated annealing, a method that involves sequentially “heating” and ‘cooling’ the system to increase the chances of overcoming energy barriers by temporarily accepting energetically unfavorable moves. This approach enhances the efficiency of sampling and optimization in complex systems, making MC simulations a versatile and valuable tool in the study of viral capsid assembly and other biologically significant processes. J. Andrew McCammon, Deqiang Zhang, and their collaborators in 2004 modeled the Cowpea Chlorotic Mottle Virus (CCMV) using a Monte Carlo method and a CG approach to investigate electrostatic interactions between RNA and the capsid [73]. In 2010, Iain G. Johnston, Ard A. Louis, and Jonathan P. K. Doye used Monte Carlo simulations to study a coarse-grained model, originally proposed by Wales, for the reversible and monodisperse self-assembly of icosahedral virus capsids. The model successfully replicates experimental features like sigmoidal assembly dynamics, hysteresis, and kinetic traps, and shows that macromolecular crowding can alter capsid yields, decreasing them under optimal conditions but potentially increasing them in suboptimal regimes. This study also highlights the value of combining computational methods to enhance our understanding of viral capsid assembly and complement experimental findings [74]. Such validations not only strengthen the credibility of computational models but also enhance their utility in drug discovery and the design of antiviral strategies. The integration of computational results with experimental validation is essential for advancing our understanding of viral capsid assembly and guiding practical applications.

Brownian dynamics (BD) is closely related to Langevin dynamics and can actually be seen as an application of the Langevin equation in specific situations, which used to model the motion of particles suspended in a fluid, taking into account the random collisions they experience with surrounding solvent molecules. The method is particularly effective for studying the behavior of large biomolecules, such as proteins, nucleic acids, and polymers, in solution. Unlike molecular dynamics (MD), which tracks the detailed motion of atoms by calculating all interatomic forces, BD simplifies the problem by focusing on the diffusion and drift of particles influenced by thermal noise and frictional forces. The core of BD lies in the Langevin dynamics, where the deterministic forces (such as those from molecular interactions) are combined with stochastic forces that represent random collisions with the solvent molecules. This allows BD to efficiently model the long-term behavior of systems, capturing the dynamics of processes that occur over extended timescales, such as protein folding, molecular self-assembly, and the movement of colloidal particles. By reducing the computational complexity while still accurately representing the essential physical processes, BD provides a valuable tool for exploring the kinetic behavior of complex biological and soft matter systems. Michael F. Hagan collaborated with fellow researchers on numerous studies utilizing Brownian Dynamics (BD) simulations to investigate virus capsid assembly. In 2006, M. F. Hagan and D. Chandler conducted a series of BD simulations on collections of bacteriophage capsids. They examined how the forces driving assembly affect the structure and the rate of capsid assembly by simulating assembly for various system parameters [75]. Their further studies extended these simulations to treat capsid assembly around icosahedral morphologies and nanoparticle cargoes of varying sizes [76,77]. In 2010, Hagan and Elrad investigated the lag phase in the kinetics of viral protein assembly using BD simulations. They determined that the critical nucleus size could be identified from the concentration dependence of the assembly half-life, and the elongation time was indicated by the length of the lag phase [78]. In 2015, Hagan and J. D. Perlmutter used BD simulations to investigate factors influencing the efficiency and specificity of assembly, including solution conditions and the quantity and potency of packaging sites. They combined a coarse-grained particle-based model for capsid proteins and RNA, investigating protein-RNA interactions arising from nonspecific electrostatics and specific packaging site interactions [79]. In 2016, Hagan and Guillermo R. Lazaro conducted a comprehensive investigation into the influence of allostery on the assembly of icosahedral shells. Their study, using BD simulations incorporating conformational dynamics, revealed that allostery significantly shifts the range of protein binding affinities crucial for successful assembly [80]. Hagan’s lab also simulated the assembly dynamics of icosahedral shells, classifying assembly pathways that lead to the encapsulation of many-molecule cargoes using the Langevin dynamics algorithm in HOOMD. Their simulations predicted intermediates and closure mechanisms not accessible through experiments [81]. In 2023, Hagan, Uri Raviv, and their coworkers used Langevin dynamics simulations with a coarse-grained model to reveal the structures of virus-like particles (VLPs), which are noninfectious nanocapsules used for drug delivery or vaccine applications. They determined the effect of ionic strength on the assembly of Simian Vacuolating Virus 40 (SV40)-like particles [82].

Overall, the significance of stochastic simulation methods in handling complex systems is reflected in their ability to explore areas that traditional methods cannot easily reach, providing deep insights into system behavior and guiding further experimental and theoretical research. This makes stochastic simulation methods an indispensable tool in modern scientific research. The studies for integrating stochastic dynamics simulations with other computational techniques, enhancing our understanding of the complex mechanisms driving viral capsid assembly.

## 4. Coarse-Grained Models

**Figure 2:**
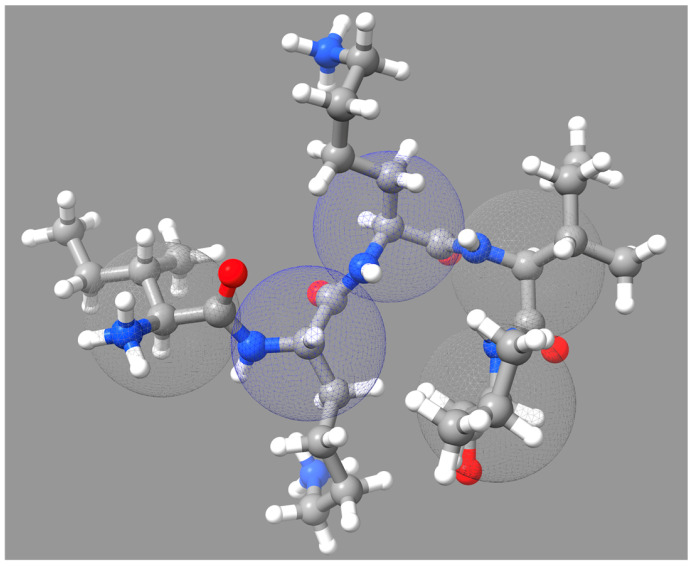
The schematic illustration of the coarse-grained models.

Coarse-grained (CG) models, also called united atom models, are a way to study the dynamics of virus capsid assembly by simplifying the system at hand and reducing computational complexity. Instead of representing every atom, CG models group atoms into larger particles, allowing the simulation of larger systems and longer timescales than all-atom models. The schematic illustration of the coarse-grained models is shown in Figure 2. This approach is particularly advantageous for capturing large-scale conformational changes and interactions in capsid assembly, providing a balance between computational efficiency and structural detail. A variety of CG protein models have been developed in recent years. Different laboratories design diverse CG models based on the knowledge of the target protein structure and the purpose of the modeling study. Coarse-grained models can mimic assembly pathways quite well. They provide mechanistic insights into the virus assembly process. The main advantage of CG models is their ability to simulate complex processes over biologically relevant timescales, which is often unfeasible with all-atom simulations due to computational constraints. However, this simplification can also be a disadvantage, as it may overlook critical atomic-level interactions and require careful parameterization to ensure accuracy. The development of the MARTINI force field made coarse-grained simulations more efficient and feasible in biomolecular research [83,84,85]. The force field simulates the behavior of biomolecular systems by combining groups of atoms in molecules into coarse-grained beads. It is suitable for simulating large molecular systems such as proteins, lipids, nucleic acids and their interactions. In the study of viral capsid assembly, the application of the MARTINI force field allows researchers to simulate the assembly process of viral protein subunits without having to deal with the detailed information of each atom.

Research on viral capsid assembly using coarse-grained (CG) models and related computational approaches has seen significant advancements over the years. Early efforts focused on modeling in vitro experiments where pure proteins self-assemble into empty capsids. For instance, in 2004, Wales developed a coarse-grained model that revealed how a thermodynamically stable and kinetically accessible icosahedral shell forms when pentameric building blocks have an optimal shape, i.e., neither too flat nor too spiky. This model highlighted that the structure of the potential energy surface (PES) could be a common feature across various systems where directed searches circumvent Levinthal’s paradox, such as in magic number clusters, protein folding, and crystallization [86].

Building on these foundational studies, recent research has incorporated more complex environmental factors, such as molecular crowding and membrane effects, into CG models. For example, in 2013, Christos Likos and R. Matthews used CG models to explore how a fluctuating fluid membrane impacts the dynamics of patchy-particle assembly into virus-like capsid cores, underscoring the influence of environmental conditions on assembly dynamics [87]. The following year, Russell Schwartz’s lab integrated particle simulation methods with CG stochastic models to investigate how molecular crowding affects capsid assembly pathways, providing valuable insights into RNA’s role in these processes and aligning simulation results with in vitro data [88,89]. A pivotal study by Perlmutter and Hagan in 2015 utilized CG simulations in combination with Brownian dynamics (BD) simulations to explore the role of packaging signals in the efficient and specific assembly of viral capsids, particularly emphasizing the critical interactions between RNA and proteins [79].

Some work further expanded the applications of CG models, Gregory A. Voth’s lab in 2017 developed a model based on sub-nanometer resolution structural data, which elucidated the interaction network regulating the early stages of HIV-1 assembly and budding [90]. By 2019, Robijn Bruinsma and colleagues used a CG model to replicate the protracted pausing and stalling observed in the budding process of alphaviruses, offering a kinetic perspective on these phenomena [91]. The following year, Jonathan P. K. Doye’s team employed CG simulations to study the reversible and monodisperse self-assembly of simple icosahedral virus capsid structures, successfully capturing many features observed in experiments [74]. Also in 2020, Dr. Gregory A. Voth’s lab revealed the dynamic mechanisms of lattice self-assembly induced by TRIM5α oligomerization, utilizing CG molecular dynamics simulations combined with electron cryo-tomography imaging [92]. HV Guzman’s group further advanced the field by proposing a methodology that combines CG models with Poisson–Boltzmann simulations to analyze viral capsid disassembly from a free energy perspective [93]. In 2021, Horacio V. Guzman and Simón Poblete introduced a CG model combined with a subdomain composition scheme, offering a comprehensive reconstruction of RNA genomes in 3D, thus enhancing our understanding of viral genome organization [94]. Most recently, in 2022, Michael F. Hagan’s team utilized a CG model to describe HBV capsid assembly and its dimorphism, successfully replicating experimental findings related to HBV assembly pathways and outcomes [95]. These computational approaches have provided detailed predictions of molecular interactions, structural dynamics, and assembly mechanisms, significantly enriching our understanding of viral capsid assembly. Importantly, these findings have been validated and expanded through complementary experimental techniques, such as binding assays, structural studies, and biophysical experiments, further confirming their accuracy and applicability.

In addition to these research advancements, several reviews have critically examined the role of CG simulations in understanding viral capsid assembly. In 2009, researchers at Rutgers University reviewed the energetics, thermodynamics, and assembly mechanisms of unenveloped viruses, focusing specifically on DNA virus assembly using CG models to provide a molecular-level perspective on these processes [96]. In 2011, the Mustafa Burak Boz group summarized various molecular mechanics methods, including both all-atom and CG approaches, that have been employed to investigate viral structure and assembly [97]. Furthering this discourse, in 2016, Roya Zandi and Michael F. Hagan reviewed the latest developments in CG modeling of virus assembly, discussing both advancements and applications within the field [98]. Szilard N. Fejer also contributed to the literature by reviewing CG models that replicate the basic physics of self-assembly in empty viral capsids [99]. These reviews have provided comprehensive insights into the evolving role of CG simulations in viral research, highlighting their growing importance in elucidating the fundamental principles of viral capsid assembly.

## 5. Electrostatic Analysis Approaches

**Figure 3:**
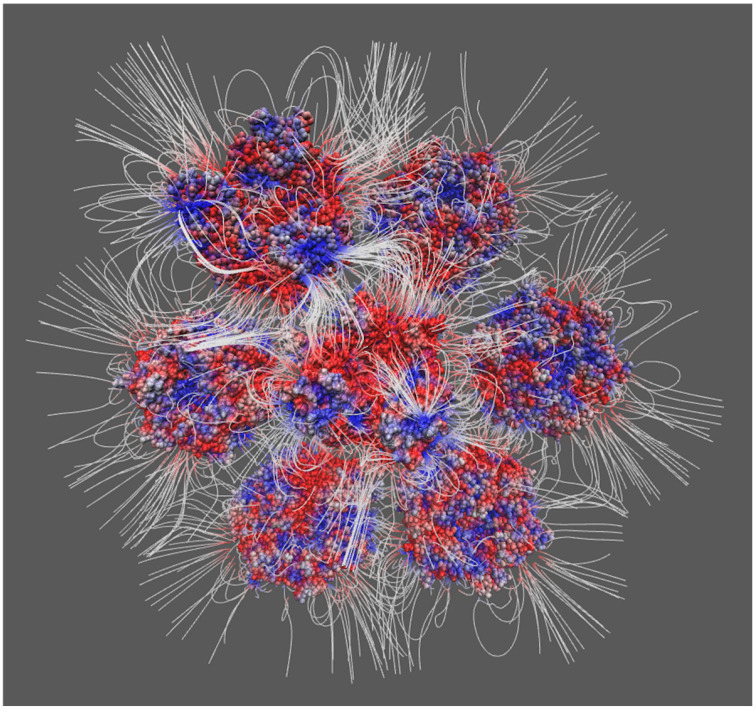
The schematic illustration of electric field lines at the binding interfaces of capsomers. The density of the electrostatic field lines indicates the strength of the electrostatic interactions between capsomers.

Electrostatic analysis methods are integral to the study of viral capsid assembly, playing a critical role in understanding the electrostatic interactions that drive the assembly process. The core of these methods involves solving the Poisson–Boltzmann equation:

(5) $$\nabla \cdot \left[\epsilon \left(r\right)\nabla \phi \left(r\right)\right]=-4\pi \rho \left(r\right)+\epsilon \left(r\right)\kappa^{2}\left(r\right)sinh\left(\frac{\phi \left(r\right)}{k_{B}T}\right),$$

where $\phi \left(r\right)$ is the electrostatic potential, $\rho \left(r\right)$ is the charge density, $\epsilon \left(r\right)$ is the dielectric permittivity, κ is the Debye–Hückel parameter, $k_{B}$ is the Boltzmann constant, and T is temperature. DelPhi [100] is a widely used computational tool for solving the Poisson–Boltzmann equation, which calculates electrostatic potentials within biological macromolecules, such as proteins and nucleic acids, in a solvent. DelPhiForce [101,102] extends this capability by not only computing potentials but also by calculating the forces between interacting proteins or between proteins and nucleic acids based on these potentials. These tools are essential for understanding the electrostatic contributions to molecular interactions, stability, and assembly processes in complex biological systems [4,53,103,104,105]. One key application of these methods is to calculate the electrostatic potential distribution around proteins and nucleic acids, using electrostatic potential maps to visually represent the charge distribution on molecular surfaces. One key application of these methods is to calculate the electrostatic potential distribution around proteins and nucleic acids, using electrostatic potential maps to visually represent the charge distribution on molecular surfaces. These analyses allow for the identification of key charged residues and electrostatic hotspots that are crucial in driving the assembly process, offering valuable insights into how variations in ionic strength and pH can influence capsid stability and formation. For instance, Figure 3 illustrates the application of electrostatic analysis in studying protein–protein interactions during viral capsid assembly. Despite its strengths, such as the ability to pinpoint crucial electrostatic interactions, the approach can be computationally intensive, especially for large systems. Moreover, it often requires simplifications that might overlook complex interactions, necessitating the integration of other computational methods to achieve a more comprehensive understanding. However, electrostatic analysis also has certain limitations, such as potential inaccuracies in handling multi-body interactions or high ionic strength environments. To address these challenges, it is often necessary to integrate other computational approaches, such as molecular dynamics simulations and more advanced numerical techniques, to enhance accuracy and reliability. Despite these challenges, electrostatic analysis methods remain invaluable in uncovering the mechanisms of viral assembly, modeling assembly pathways, and designing strategies to inhibit viral capsid formation.

Electrostatic interactions have long been recognized as crucial in the process of viral capsid assembly and maturation. As early as 1994, John E. Johnson’s lab employed electrostatic methods alongside molecular genetic studies to investigate the maturation of noninfectious nodavirus provirions’ icosahedral capsids, focusing on the role of a critical aspartic acid residue in the autoproteolytic cleavage process. Their findings underscored the significant role of electrostatic interactions in capsid maturation, particularly highlighting the unique involvement of a single acidic residue in the hydrolytic cleavage of a peptide bond, a mechanism that contrasts with the traditional involvement of two acidic residues in acid proteases [106]. Building on this foundation, in 1998, Charles L. Brooks III, John E. Johnson, and their collaborators further explored the role of electrostatics by calculating association energies for specific subunit interfaces across three distinct icosahedral viruses. This study, leveraging atomic resolution structures combined with molecular mechanics and continuum electrostatic methods, provided a sensitive approach to identifying unique intermediates and pathways in virus capsid assembly [107]. In recent years, there has been a notable surge in research utilizing electrostatic analysis approaches to deepen our understanding of viral capsid assembly. For example, J. Andrew McCammon and Deqiang Zhang explored the electrostatic interactions between RNA and the capsid using a Monte Carlo method combined with a coarse-grained (CG) approach, where electrostatic potentials were calculated through Poisson–Boltzmann equations [73]. Similarly, Robert Konecny and his colleagues studied the electrostatic properties of Cowpea Chlorotic Mottle Virus (CCMV) and Cucumber Mosaic Virus (CMV) by solving the Poisson–Boltzmann equation, further elucidating the role of electrostatics in viral stability [108]. In 2010, A. G. Cherstvy reviewed the charge effects in biological DNA-related systems, emphasizing the importance of electrostatic interactions in DNA spooling within viral capsids [109]. Further advancing the field, Roya Zandi and her team, in 2014, investigated the pivotal electrostatic interactions between RNA and the charged inner capsid wall, which are critical in the spontaneous co-assembly of the genome and capsid proteins. Using a field theoretic formulation, they demonstrated that the branched RNA secondary structure plays a significant role in optimizing the encapsulated genome volume and enhancing assembly efficiency in viruses [110]. In 2019, Lin Li, Chuan Xiao, and their research team used multi-scale computational approaches, including DelPhi, DelPhiForce, molecular dynamics (MD) simulations, and MM/PBSA, to investigate the electrostatic interactions in the capsid assembly mechanisms of the giant virus Paramecium bursaria chlorella virus-1 (PBCV-1) [10]. The same year, Lin Li’s lab also examined the interactions between dengue heterotetramers and their roles in capsid assembly using MD simulations and electrostatic analysis [65]. Further highlighting the importance of electrostatic analysis, in 2020, HV Guzman’s group proposed a methodology that combines coarse-grained models with Poisson–Boltzmann simulations to analyze the viral capsid disassembly process from a free energy perspective. This study provided a comprehensive understanding of the disassembly mechanism by considering the effects of pH and the charge of the genetic material inside the capsid. They found that while an alkaline environment enhances the stability of the Triatoma virus (TrV) capsid, the resulting deprotonation of the genetic material generates a Coulomb-type electrostatic repulsion that triggers disassembly [93]. In conclusion, these studies underscore the critical role of electrostatic interactions in viral capsid assembly and maturation. They also demonstrate how electrostatic analysis, through advanced computational methods, can unravel the complexities of viral structure and function, providing insights that are essential for developing antiviral strategies and understanding viral evolution.

## 6. Other Computational Models

In addition to molecular dynamics (MD), stochastic dynamics simulations, coarse-grained (CG) models, and electrostatic analysis, several other computational approaches have been utilized to study viral capsid assembly. These approaches include lattice models, hybrid methods, machine learning techniques and kinetic models. Lattice models simplify the spatial complexity of capsid assembly, allowing researchers to investigate the effects of geometric constraints and symmetry on the assembly process. Hybrid methods, which combine different computational techniques, offer a more comprehensive understanding by integrating the strengths of various approaches, such as the detailed atomic resolution of MD with the broader statistical insights provided by stochastic dynamics simulations. Machine learning techniques are increasingly being applied to predict assembly pathways and identify key factors influencing assembly efficiency, leveraging large datasets generated from experiments and simulations. Kinetic models provide insights into the rates of assembly steps and the identification of rate-limiting stages, which are crucial for understanding and optimizing the efficiency of the overall capsid assembly process. The overarching goal across these methods is to develop the simplest model that remains consistent with experimental data, thereby uncovering fundamental insights into the principles governing capsid assembly and informing the design of antiviral strategies and nanotechnology applications.

Among the various methods used to study viral capsid assembly, lattice models hold a significant position, particularly in exploring and understanding the fundamental mechanisms of the assembly process. Lattice models simplify the system by representing capsid proteins on a discrete lattice, allowing for the exploration of general assembly principles and the identification of potential assembly pathways. Compared to methods like molecular dynamics (MD) simulations and coarse-grained (CG) models, lattice models simplify spatial and molecular details, yet they provide in-depth insights into assembly pathways, dynamic behavior, and geometric constraints at a lower computational cost. These models are particularly well-suited for large-scale parameter scanning and analyzing assembly efficiency under different conditions, helping researchers uncover the regularities and potential control mechanisms of viral capsid self-assembly. When combined with other methods, lattice models offer a powerful tool for studying viral capsid assembly, enabling the capture of system behavior on a larger scale while providing a theoretical foundation for more detailed model studies. The advantage of lattice models lies in their computational efficiency and simplicity, making them suitable for studying large systems over long timescales. However, these models often lack the detailed atomic interactions provided by other methods. This may limit their predictive accuracy for real biological systems. For example, Timothy S. Baker and Robert S. Sinkovits derived a complete set of rules for constructing icosahedral structures from symmetrons when the T lattice symmetry is odd in 2010. Their work demonstrated the existence of three classes of solutions, which encompass all conceivable methods of constructing an icosahedral structure using only trisymmetrons and pentasymmetrons [111].

Hybrid methods, which combine different computational approaches, play a crucial role in viral capsid assembly research by leveraging the strengths of each method. For example, a common hybrid approach integrates coarse-grained (CG) models with all-atom molecular dynamics (MD) simulations, balancing computational efficiency with the need for detailed accuracy. This combination allows researchers to study large, complex systems while retaining the ability to zoom in on specific regions for in-depth analysis. However, integrating different methods can be complex and computationally demanding, and balancing resolution and accuracy across different scales presents significant challenges. Numerous studies have employed hybrid methods to reduce the computational costs associated with all-atom simulations while maintaining a high level of detail. For instance, in 2006, Wah Chiu and colleagues developed a hybrid modeling approach that integrated CryoEM density data with ab initio modeling techniques to generate a structural model for the core domain of a herpesvirus structural protein, VP26 [112]. This approach has since been applied to modeling various components of large macromolecular assemblies. Similarly, in 2009, Stephen C. Harvey’s group used a hybrid method to develop an all-atom model of the Pariacoto Virus (PaV) by combining initial CG simulations with all-atom models, providing detailed structural and electrostatic characterizations of viral assembly [113]. In 2020, Meera Sitharam’s lab identified critical inter-atomic hotspot interactions in the virus assembly process. They combined a geometric method for rapidly plotting free energy landscapes with a symmetry-based combinatorial approach to predict hotspot interactions for adeno-associated virus serotype 2 (AAV2), minute virus of mice (MVM), and bromo mosaic virus (BMV) [114]. These examples underscore the versatility and power of hybrid methods in addressing the complexities of viral capsid assembly, allowing for both broad-scale analysis and detailed structural insights.

Machine learning (ML) techniques, particularly deep learning-based AI methods, have recently emerged as transformative tools for modeling viral capsid assembly pathways and optimizing antiviral strategies. By analyzing large datasets from experimental and simulation studies, these advanced ML models can identify patterns and make predictions about assembly mechanisms that traditional methods might overlook. The primary strength of deep learning lies in its ability to process and analyze complex, high-dimensional data, offering novel insights into the factors driving capsid assembly processes. However, the effectiveness of these models heavily relies on the quality and quantity of the training data, and their interpretability remains a significant challenge. Recent advancements emphasize the potential of ML in this domain. For example, a 2021 study by Sergei Zolotukhin et al. employed artificial neural networks (ANNs) and support vector machines (SVMs) to analyze next-generation sequencing data from complex adeno-associated virus (AAV) capsid libraries. This approach accurately predicted viable capsid variants and identified critical mutation patterns, offering a framework for guiding viral vector design [115]. Similarly, deep learning models have been utilized to design highly diverse and viable Adeno-associated Virus 2 (AAV2) capsid protein variants, surpassing natural sequence diversity and unlocking new areas of sequence space for improved viral vectors and protein therapeutics [116]. In 2022, Antoni Luque’s group further advanced the field by combining ML with an allometric model to predict tailed phage capsid architectures based on the major capsid protein gene. Their findings revealed diverse capsid sizes in human gut metagenomes, offering valuable insights into capsid evolution and selection in ecosystems [117]. These studies exemplify the power of ML techniques not only in advancing our understanding of viral capsid assembly but also in driving innovation in antiviral strategies, viral vector development, and therapeutic protein design. The integration of ML into these areas represents a critical step forward in tackling challenges in virology and therapeutic development.

Kinetic models are essential computational tools for describing and predicting the time-dependent processes involved in molecular interactions and assembly, with a particular focus on the rates at which these processes occur. In the study of viral capsid assembly, kinetic models are crucial for understanding how protein subunits dynamically come together to form a complete capsid. These models enable researchers to simulate assembly pathways, identify key intermediate states, and determine the rate-limiting steps that govern the efficiency and fidelity of the assembly process. By fitting kinetic models to experimental data, such as rate constants obtained from in vitro assembly studies, scientists can gain valuable insights into the factors that influence capsid formation. This knowledge is pivotal for developing antiviral strategies aimed at disrupting the assembly process, as well as for designing synthetic capsids for biomedical applications. For instance, in 2017, Russell Schwartz’s laboratory optimized the rate parameters of capsid assembly models to better align kinetic rate constants with experimental data, enhancing the predictive accuracy of these models [118]. Building on this, in 2018, Schwartz and M. Senthil Kumar developed a computational strategy to deduce coat-coat binding rate parameters for viral capsids by fitting stochastic simulation results to experimental observations [119]. Additionally, Robijn Bruinsma, Joseph Rudnick, and Inbal Mizrahi employed a statistical mechanics approach to elucidate the kinetic selection process of viral RNA molecules during the nucleation stage of assembly in small RNA viruses, further demonstrating the power of kinetic models in advancing our understanding of viral assembly mechanisms [120].

Various computational methods have significantly contributed to advancing our understanding of viral capsid assembly by addressing different aspects of the process. Techniques such as rigid-cluster elastic network interpolation, ordinary differential equation models, stochastic discrete event simulations, and energy-landscape-based methods have provided insights into transition pathways, assembly mechanisms, and structural stability. Additionally, innovative approaches like self-consistent field theory, graph-theoretical analyses, and statistical ensemble-based models have further refined our ability to predict and analyze capsid behavior under different conditions. Collectively, these methods complement traditional approaches, offering a deeper and more comprehensive understanding of the intricate dynamics involved in viral capsid assembly.

We have summarized several popular software tools for molecular dynamics (MD), Monte Carlo (MC), coarse-grained (CG), and electrostatic analysis (Table 1). These tools represent the forefront of computational modeling and are widely used in diverse scientific fields, including structural biology, materials science, and biophysics. For molecular dynamics, there are many software options available because MD simulations are widely used to study biomolecular systems. Their popularity comes from their broad applications and the ongoing development of methods to handle more complex systems. Monte Carlo and coarse-grained simulations, while important for tasks like probabilistic modeling or studying large systems, have fewer standalone tools. This is because these methods are often included as features within larger simulation platforms, such as MD software or hybrid frameworks, rather than existing as separate programs. Electrostatic analysis tools are essential in biomolecular modeling for understanding interactions and energies. These tools are important for complementing MD and CG studies. While this list is not exhaustive, it highlights some of the most commonly used tools in each category to provide a comprehensive resource for researchers selecting the appropriate computational methods for their studies.

## 7. Conclusions

In conclusion, a variety of computational methods have significantly advanced our understanding of viral capsid assembly, each offering unique advantages and facing specific challenges. Molecular dynamics (MD) simulations provide detailed atomic-level insights but are limited by high computational costs and short simulation timescales. MC simulations efficiently explore the stochastic nature of assembly processes but often lack detailed temporal information. Coarse-grained (CG) models strike a balance between computational efficiency and structural detail, although they require careful parameterization to ensure accuracy. Electrostatic analysis has highlighted the crucial role of electrostatic interactions in capsid assembly, identifying key charged residues and electrostatic hotspots. However, the computational intensity of this approach, particularly for large systems, poses significant challenges. Other methods, such as lattice models, hybrid approaches, and machine learning techniques, complement traditional approaches by offering new perspectives and predictive capabilities.

Different viruses employ distinct assembly strategies, reflecting the diversity of their structures and life cycles, which have significant implications for the choice of appropriate assembly models. For example, some viruses, such as bacteriophages, often assemble a preformed capsid, known as a procapsid, which is then filled with genetic material by a motor-like mechanism. In contrast, many RNA viruses, like the poliovirus, co-assemble their capsid proteins directly around their RNA genome, ensuring that the genetic material is encapsulated as the capsid forms. Additionally, some viruses, such as retroviruses like HIV, utilize a nucleocapsid protein to assemble around their RNA genome, which is then incorporated into a viral capsid as it buds from the host cell membrane. These varying strategies highlight the adaptability of viruses to their environments and the specific requirements of their replication cycles, ultimately influencing how they interact with host cells and how they can be targeted by antiviral therapies. The diversity in these assembly strategies necessitates different computational models, such as coarse-grained MD simulations or all-atom simulations, each offering unique advantages depending on the specific viral assembly process being studied. Understanding these varied strategies is crucial for selecting the appropriate computational approach.

In recent years, advancements in algorithms have significantly enhanced the efficiency and accuracy of viral capsid assembly simulations. For instance, John M. A. Grime and Gregory A. Voth designed an efficient parallelization scheme that leverages GPU computing over traditional CPU processing, accelerating rigid body dynamics simulations [121]. This approach is particularly well-suited for handling simulations of spatially heterogeneous particle concentrations, especially during assembly processes involving implicit solvents. Additionally, enhanced sampling methods have further improved the efficiency and precision of these simulations. The future of computational virology lies in developing hybrid frameworks that combine multiple approaches to enhance scalability and accuracy, enabling the study of larger systems and more realistic simulations. Simplified models play an important role in this effort by reducing computational complexity and providing insights into large-scale systems or long-timescale processes. While they enhance efficiency, their simplified representations frequently compromise accuracy, potentially overlooking critical molecular interactions, dynamic assembly pathways, or non-linear effects. For example, coarse-grained models may miss atomic-level details crucial for specific binding interactions, while static approximations might fail to capture intermediate states in dynamic processes. Multi-scale modeling approaches, which combine simplified and high-resolution models, offer a way to balance accuracy and efficiency. Advances in algorithms and GPU-accelerated methods, as well as data-driven techniques like machine learning, are further improving the predictive power of simplified models. Ensuring accurate parameterization and validation against experimental data, along with ad-dressing environmental factors such as solvent effects, ionic strength, and pH, remains essential for realistic and reliable simulations. Together, these advancements pave the way for more holistic and accurate approaches in computational virology.

By addressing these challenges and continuing to refine these computational approaches, researchers can develop more accurate and holistic models of viral capsid assembly. These advancements will not only deepen our understanding of the fundamental processes underlying viral assembly but will also contribute to the development of novel antiviral treatments and the design of nanomaterials for biomedical applications.

## Figures and Tables

**Table 1:** Popular software used for viral capsid assembly.

Category	Software	Authors	First Released	Features	Link
Molecular Dynamics	AMBER	Peter Kollman and David A. Case team	1978	Accurate force fields for biomolecules; excellent for proteins and nucleic acids.	https://ambermd.org (accessed on 11 December 2024)
	CHARMM	Martin Karplus and collaborators	1983	Robust force fields; supports proteins, lipids, and nucleic acids.	https://www.charmm.org (accessed on 11 December 2024)
	GROMACS	Erik Lindahl, Berk Hess, and David van der Spoel	1991	High efficiency; GPU-optimized; widely used for biomolecules.	https://www.gromacs.org (accessed on 11 December 2024)
	NAMD	Klaus Schulten team, UIUC	1995	Excellent for large-scale systems; highly parallelized; integrates with VMD.	https://www.ks.uiuc.edu/Research/namd/ (accessed on 11 December 2024)
	LAMMPS	Sandia National Laboratories	1995	Flexible and extensible; supports molecular dynamics and coarse-grained simulations.	https://lammps.org (accessed on 11 December 2024)
Monte Carlo	MCNP	Los Alamos National Laboratory	1983	Standard for particle transport simulations; widely used in nuclear physics.	https://mcnp.lanl.gov (accessed on 11 December 2024)
	Crystal Ball	Oracle company	1988	Excel-integrated; widely used for risk analysis and forecasting.	https://www.oracle.com (accessed on 11 December 2024)
	@RISK	Palisade corporation	1987	Excel-compatible; popular for probabilistic modeling and project management.	https://www.palisade.com (accessed on 11 December 2024)
Coarse-Grained	MARTINI	Marrink, Risselada, and Mark	2004	Popular for lipid and protein simulations; supports biomembranes.	http://cgmartini.nl (accessed on 11 December 2024)
	HOOMD-blue	Glotzer Lab, University of Michigan	2007	GPU-optimized; ideal for soft matter and polymer simulations.	https://glotzerlab.engin.umich.edu/hoomd-blue/ (accessed on 11 December 2024)
	CafeMol	Kazuhiro Kinoshita and collaborators	2007	Protein and RNA simulations; user-friendly for coarse-grained models.	https://www.cafemol.org (accessed on 11 December 2024)
Electrostatic Analysis	DelPhi	Honig Lab, Columbia University	1990	Accurate electrostatic potential calculations; widely used for biomolecular systems.	http://compbio.clemson.edu/lab/delphisw/ (accessed on 11 December 2024)
	APBS	Baker Lab, University of Washington	2001	Solves Poisson–Boltzmann equations; supports biomolecular electrostatics.	https://apbs.org (accessed on 11 December 2024)
	PyMOL (APBS Plugin)	Schrodinger, LLC	2000	Easy visualization of electrostatic potentials on molecular surfaces.	https://pymol.org/ (accessed on 11 December 2024)
	PMEPRO	Joint Center for Structural Genomics	2008	High-quality visualizations of protein and nucleic acid electrostatics.	http://www.jcsg.org (accessed on 11 December 2024)

## Data Availability

This review article does not present original data or new software. All data discussed and cited within the review are derived from previously published studies, which are appropriately referenced. Any relevant datasets or software tools used in the reviewed studies can be accessed through the corresponding references provided in the text.
